# Monitoring Local Regional Hemodynamic Signal Changes during Motor Execution and Motor Imagery Using Near-Infrared Spectroscopy

**DOI:** 10.3389/fphys.2015.00416

**Published:** 2016-01-11

**Authors:** Naoki Iso, Takefumi Moriuchi, Akira Sagari, Eiji Kitajima, Fumiko Iso, Koji Tanaka, Yasuki Kikuchi, Takayuki Tabira, Toshio Higashi

**Affiliations:** ^1^Unit of Rehabilitation Sciences, Nagasaki University Graduate School of Biomedical SciencesNagasaki, Japan; ^2^Medical Corporation Toujinkai Miharadai HospitalNagasaki, Japan; ^3^Japanese Red Cross Society Nagasaki Genbaku HospitalNagasaki, Japan; ^4^Center for Industry, University and Government Cooperation, Nagasaki UniversityNagasaki, Japan; ^5^Unit of Physical and Occupational Therapy, Nagasaki University Graduate School of Biomedical SciencesNagasaki, Japan; ^6^Department of Occupational Therapy, Faculty of Rehabilitation Sciences, Nishikyushu UniversitySaga, Japan

**Keywords:** motor imagery, mental practice, motor execution, NIRS, hemodynamic signal changes

## Abstract

The aim of this study was to clarify the topographical localization of motor-related regional hemodynamic signal changes during motor execution (ME) and motor imagery (MI) by using near-infrared spectroscopy (NIRS), as this technique is more clinically expedient than established methods (e.g., fMRI). Twenty right-handed healthy subjects participated in this study. The experimental protocol was a blocked design consisting of 3 cycles of 20 s of task performance and 30 s of rest. The tapping sequence task was performed with their fingers under 4 conditions: ME and MI with the right or left hand. Hemodynamic brain activity was measured with NIRS to monitor changes in oxygenated hemoglobin (oxy-Hb) concentration. Oxy-Hb in the somatosensory motor cortex (SMC) increased significantly only during contralateral ME and showed a significant interaction between task and hand. There was a main effect of hand in the left SMC. Although there were no significant main effects or interactions in the supplemental motor area (SMA) and premotor area (PMA), oxy-Hb increased substantially under all conditions. These results clarified the topographical localization by motor-related regional hemodynamic signal changes during ME and MI by using NIRS.

## Introduction

Recent studies have shown that mental practice in which motor imagery (MI) is performed repeatedly can improve motor functions in patients after stroke; these effects have been demonstrated in clinical studies using randomized controlled trials (Page et al., 2001, 2012; Liu et al., 2004; Sharma et al., 2006; Riccio et al., 2010). An important aspect in mental practice is how vividly an individual can perform MI. It has been reported that the vividness of MI depends on the experience in performing the task (Mulder et al., 2004), contents of the task, and individual's ability for imagery (Schuster et al., 2011). The vividness of MI can be evaluated using questionnaires such as the Kinesthetic and Visual Imagery Questionnaire-20 (KVIQ-20) (Malouin et al., 2007) and the Movement Imagery Questionnaire-Revised Second Version (MIQ-RS) (Gregg et al., 2010). However, as these are subjective evaluations, they do not provide sufficient confirmation of the vividness of MI. Objective methods to assess the vividness of MI effectively in a clinical setting have not been established yet.

The difference between the regions activated during MI and motor execution (ME) has been assessed in different studies by applying a wide variety of techniques. These studies have reported that brain activation during MI is mostly similar to that during ME (Jeannerod, 2001; Kimberley et al., 2006). Although several studies using functional magnetic resonance imaging (fMRI) or positron emission tomography (PET) have demonstrated activation of regions related to motor execution such as the supplementary motor area (SMA) and premotor area (PMA) during MI (Stephan et al., 1995; Ruby and Decety, 2003; Solodkin et al., 2004), non-activation of the primary motor cortex has been reported (Hanakawa et al., 2003; Lotze et al., 2003; Cunnington et al., 2005). In contrast, previous studies using transcranial magnetic stimulation (TMS) showed increased activation of the primary motor cortex during MI (Stinear and Byblow, 2003; Pelgrims et al., 2011). This inconsistency regarding brain activation during MI may result from differences in the time and spatial resolution of each technique.

Recently, there have been many reports of near-infrared spectroscopy (NIRS) used in the field of rehabilitation (Miyai et al., 2003; Mihara et al., 2012; Kober et al., 2014). NIRS is a non-invasive optical neuroimaging technique that measures concentration changes of oxygenated hemoglobin (oxy-Hb) in the cerebral vessels based on differences in the absorption spectra for light in the near-infrared range. This method has been promoted for its non-invasive nature, relatively low cost (Obrig and Villringer, 2003; Tobias, 2006; Steinkellner et al., 2010; Koenraadt et al., 2014), ease of integration with other modalities such as electroencephalography (Lareau et al., 2011; Fazli et al., 2012) or fMRI (Mehagnoul-Schipper et al., 2002; Cooper et al., 2012), and portability (Atsumori et al., 2009; Piper et al., 2014). Because NIRS is portable and less restrictive, it is possible to measure hemodynamic signal changes in various settings and stages of rehabilitation, even at the bedside. Accordingly, we propose that monitoring local regional hemodynamic signal change using NIRS can be applied for objective assessment of vividness of MI. For this purpose, it is necessary to clarify the topographical localization of motor-related regional hemodynamic signal changes during ME and MI by using NIRS.

However, the existing knowledge regarding hemodynamic signal changes during MI measured with NIRS is still limited. Some previous studies have already investigated hemodynamic signal changes during MI. Mihara et al. found that neurofeedback with NIRS during MI significantly induced activation of the contralateral PMA, and they suggested effectiveness of neurofeedback system using NIRS on the performance of kinesthetic MI (Mihara et al., 2012). Wriessnegger et al. provided new results concerning timing and brain oxygenation of the somatosensory area and the motor cortex during MI measured using NIRS (Wriessnegger et al., 2008). In the recent study, Kober et al. reported timing and topographical distribution of the hemodynamic response during ME and MI of swallowing (Kober and Wood, 2014). Thus, to the best of our knowledge, no study has investigated the topographical localization of motor-related regional hemodynamic signal changes using NIRS during ME and MI of finger task. Moreover, we expect that NIRS can be readily developed for studies in the clinic and other challenging settings, given the convenience of NIRS, which has exceptional portability and adaptability. In particular, NIRS can be used easily in the field of rehabilitation because it is non-invasive and minimally restrictive.

Therefore, the aim of this study was to monitor hemodynamic signal changes of regions related to motor execution with NIRS during MI and ME by using a tapping sequence task, and to clarify the topographical localization of motor-related regional hemodynamic signal changes during ME and MI using the clinically expedient method of NIRS.

## Materials and methods

### Subjects

The participants were 20 neurologically healthy right-handed adults (13 males, mean age 28 years, range 21–47 years). Handedness was determined using the Edinburgh Handedness Inventory (Oldfield, 1971); none of the participants had a history of handedness conversion. The study was approved by the local ethics committee at Nagasaki University Graduate School of Biomedical Sciences, and all participants provided written informed consent.

### Experimental procedure

**Figure 1:**
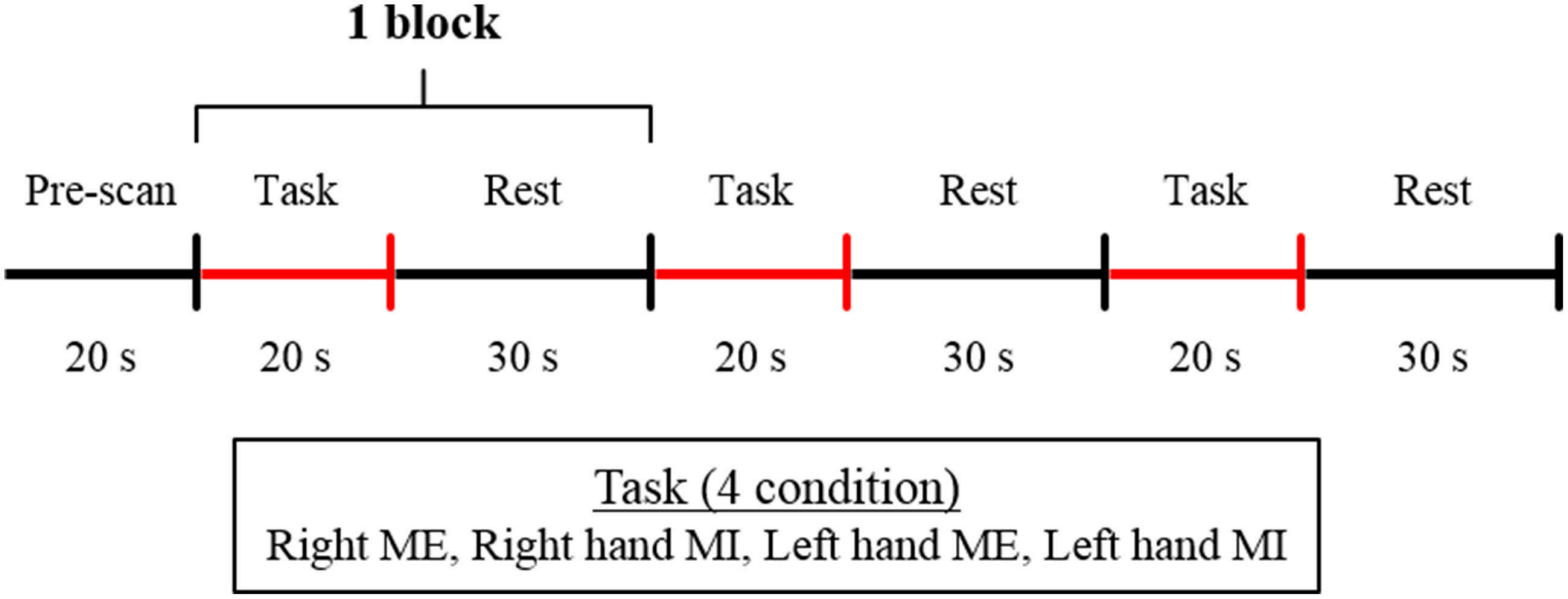
**Experimental protocol**. The blocked design consisted of 3 cycles of 20 s of task performance and 30 s of rest. The experiment was performed under 4 conditions: ME, motor execution; MI, motor imagery with the right or left hand.

The participants sat on a comfortable chair and placed their hands on a table in a pronated position. The basic task consisted of a tapping sequence used in a previous study (Roland et al., 1980). The thumb must, in quick succession, briefly touch the index finger 2 times, the middle finger once, the ring finger 3 times, and the little finger 2 times; then, with the thumb in this position, the order of movement is reversed. The tapping sequence task was performed under four conditions: ME and MI with the right and left hand; all participants performed the task under all the four conditions. The participants were instructed to perform the ME and MI task within 20 s. The experimental protocol was a blocked design that consisted of 3 cycles of 20 s of task performance and 30 s of rest in each experimental condition (Figure 1). The order of the conditions was counter-balanced. The participants were given sufficient time to practice the task so that they could perform the sequence in 20 s. The experiment was started when each participant was able to perform the task adequately. For MI, the participants were instructed to perform the task using kinesthetic MI as if they were actually performing the movement. Before the start of the experiment, to rate the vividness of the subjects' motor imagery, the subjects were asked to complete a self-evaluation test on a visual analog scale (VAS). That is, the subjects marked a location on a 100-mm horizontal line, the two ends of which were labeled “0 = None at all” and “100 = Very vivid image,” according to the vividness of the imagery they experienced (Lotze and Halsband, 2006; Ikeda et al., 2012). The experiment was started when the participants assessed that the vividness of their MI reached 80% on VAS (Ohno et al., 2011). The participants were instructed to maintain the same position and relax without thinking during the resting time. The participants kept their eyes closed during both resting and task performance. The participants were alerted with a beep sound during the experiment conforming to experimental protocol.

### NIRS measurement and analysis

**Figure 2:**
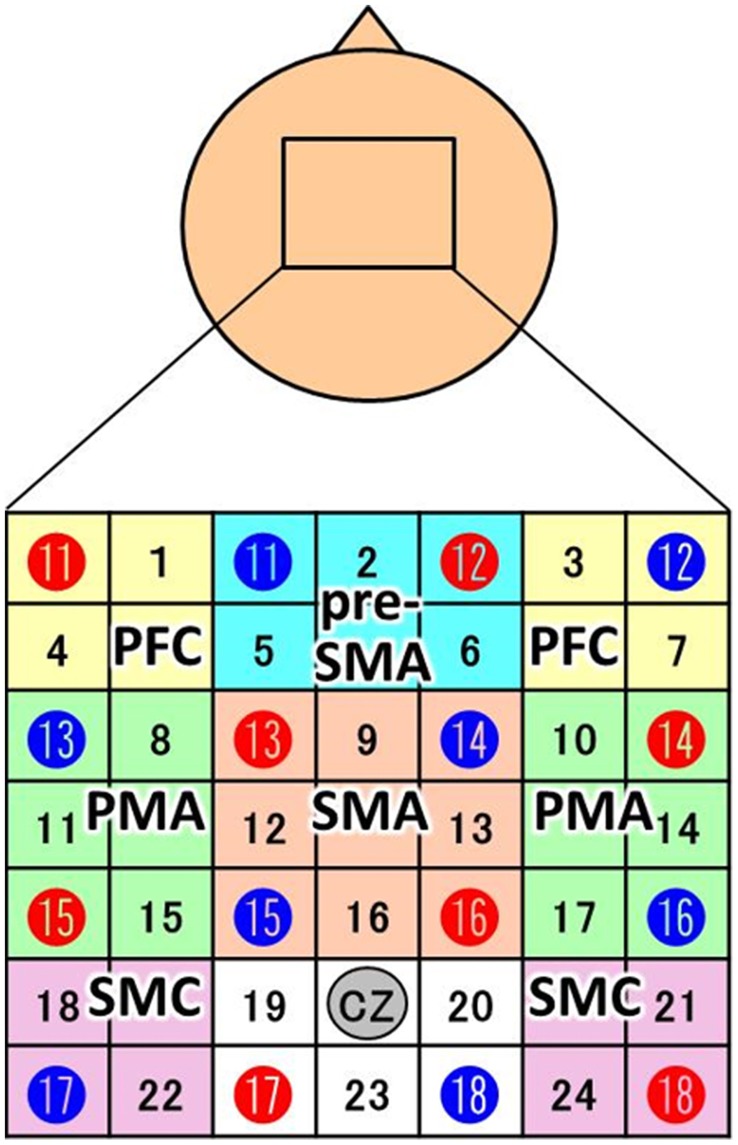
**Channel configuration of the 4 × 4 optode probe set**. The 24-channel NIRS probe set was positioned over the motor areas. Red and blue circles indicate the positions of NIRS sensors and detectors, respectively. The black numbers represent the channels, and the colored boxes show the regions of interest (PFC, prefrontal cortex; pre-SMA, pre-supplemental motor area; SMA, supplemental motor area; PMA, pre-motor area; SMC, somatosensory motor cortex). According to the international 10–20 placement system, Cz was used as a marker position to ensure replicable placement of the optodes.

NIRS measurements were performed using a continuous wave system (ETG-4000; Hitachi Medical Co., Tokyo, Japan) equipped with 4 × 4 optode probe sets (8 incident lights and 8 detector fibers), resulting in a total of 24 channels at an inter-optode distance of 3.0 cm. The NIRS channels were placed according to the international 10–20 system, and the Cz position was used as a marker for ensuring replicable placement of the optodes (Okamoto et al., 2004). The positions of the optodes were determined based on previous NIRS studies of motor-related areas (Hatakenaka et al., 2007; Amemiya et al., 2010; Sagari et al., 2015). The optodes were positioned using a custom-made cap that covered the right and left dorsolateral prefrontal cortex (PFC), pre-SMA, SMA, dorsal PMA, and somatosensory motor cortex (SMC). Regarding the SMC, the sensory area and motor cortex are often treated as a single unit in NIRS experiments (Hatakenaka et al., 2007; Amemiya et al., 2010; Sagari et al., 2015). The areas and optodes covering them were as follows: left SMC, channels 18 and 22; right SMC, channels 21 and 24; SMA, channels 9, 12, 13, and 16; pre-SMA, channels 2, 5, and 6; left PMA, channels 8, 11, and 15; right PMA, channels, 10, 14, and 17; left PFC, channels 1 and 4; and right PFC, channels 3 and 7 (Figure 2). Channels 19, 20, and 23 were not further analyzed.

**Figure 3:**
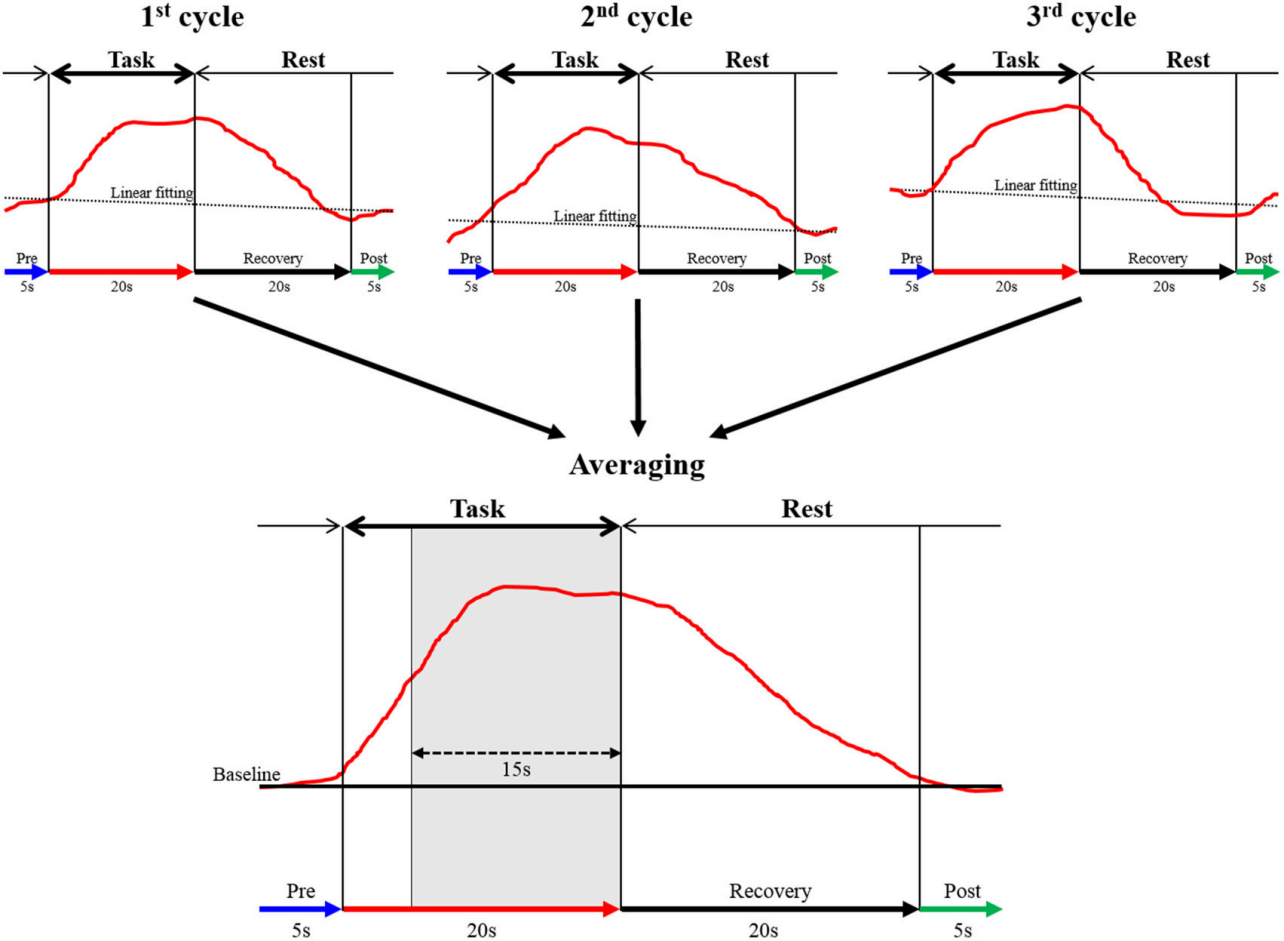
**The data analysis using integral mode**. The red curve represents the sham NIRS waveform in one case. This waveform was created by averaging the data measured over 3 cycles in a block design. Linear fitting was applied to the data between pre-task (blue arrow) and post-task (green arrow) periods. The thick black arrow above the red curve indicates the task period, and the thin arrow shows the rest period. The vertical axis represents oxy-Hb concentration (mMmm), and the horizontal axis represents the time course of 1 cycle. The mean value of oxy-Hb measured between 5 and 20 s during the task (15 s, shaded gray area in averaged graph) was calculated.

The continuous wave NIRS system uses two different wavelengths (625 and 830 nm), which were both used in this study. Relative changes in the absorption of near-infrared light were sampled at 10 Hz, and these values were converted to changes in the concentration of oxy-Hb and deoxygenated hemoglobin (deoxy-Hb) based on the modified Beer-Lambert approach (Cope and Delpy, 1988; Obrig and Villringer, 2003). In this study, we used changes in oxy-Hb concentration as an indicator of changes in regional cerebral blood volume, given that an earlier NIRS signal study of a rat brain model proposed that oxy-Hb was a sensitive parameter for brain activation (Hoshi et al., 2001). The moving average method (window: 5 s) was used to exclude short-term motion artifacts in the analyzed data. The obtained data were analyzed in the integral mode, which calculates the average waveform (Figure 3; Marumo et al., 2009; Pu et al., 2012). We determined the pre-task baseline as the mean over the 5 s prior to the task period, and the post-task baseline as the mean over the last 5 s of the post-task period. We applied for linear fitting to the data between these two baselines. We used the average waveform measured at 5–20 s after the task had started, considering the time required for changes in oxy-Hb. To assess the influence of noise, the data were high pass filtered at 3 Hz, and noise components were separated and analyzed using wave analysis. Channels whose standard deviation exceeded 0.08 were assumed to be influenced by excessive noise and were thus excluded. In addition, after removing blocks with marked body-movement artifacts, data of the remaining blocks were used. Subsequently, we calculated the mean value of oxy-Hb of each region and determined the average change in the concentration of oxy-Hb. We monitored the activation of the thenar muscles during MI by using electromyography and excluded the data when obvious muscle activation was observed.

### Statistical analysis

Two-way ANOVA was used to examine the effect of task (ME vs. MI) and hand (right hand vs. left hand) for each region of interest. The mean values for each group were compared using Bonferroni test. In addition, effect sizes were calculated. SPSS was used for statistical analysis, and statistical significance was defined as *P* < 0.05 in all cases.

## Results

**Figure 4:**
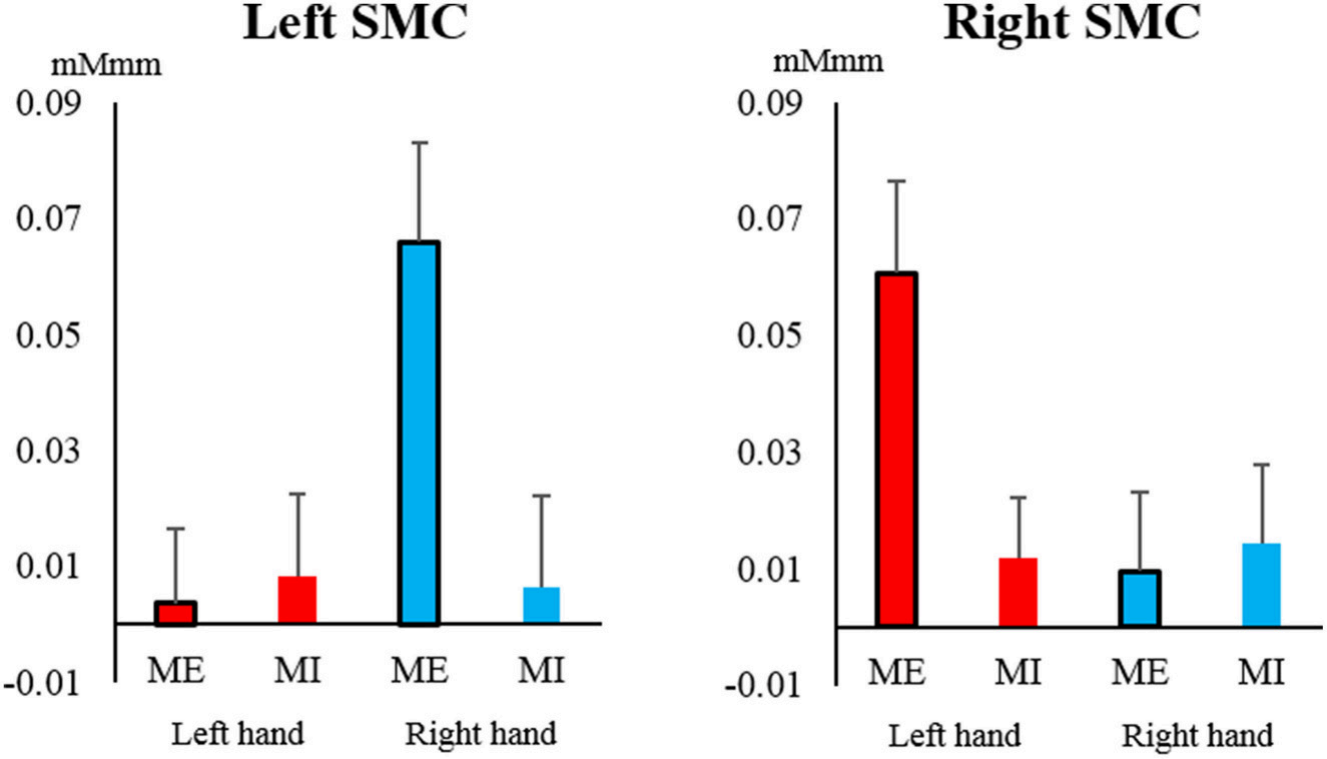
**Changes in oxy-Hb concentration during the task in the left and right somatosensory motor cortex (SMC)**. The oxy-Hb level of the SMC increased significantly only during contralateral motor execution (ME) and not during motor imagery (MI), and it showed a significant interaction between task and hand (left hand, red; right hand, blue). Vertical bars represent the standard error.

The oxy-Hb level in the SMC increased significantly only during contralateral ME and showed a significant interaction between task and hand [left SMC: *F*_(1, 19)_ = 6.749, *P* = 0.018, η^2^ = 0.058; right SMC: *F*_(1, 19)_ = 5.146, *P* = 0.035, η^2^ = 0.045] (Figure 4). A significant main effect of hand was only observed in the left SMC, indicating that the oxy-Hb level increased during ME with the right hand [hand: *F*_(1, 19)_ = 5.590, *P* = 0.029, η^2^ = 0.055; task: *F*_(1, 19)_ = 3.537, *P* = 0.075, η^2^ = 0.045], whereas none of the main effects were significant for the right SMC [hand: *F*_(1, 19)_ = 3.658, *P* = 0.071, η^2^ = 0.036; task: *F*_(1, 19)_ = 3.798, *P* = 0.066, η^2^ = 0.030].

**Figure 5:**
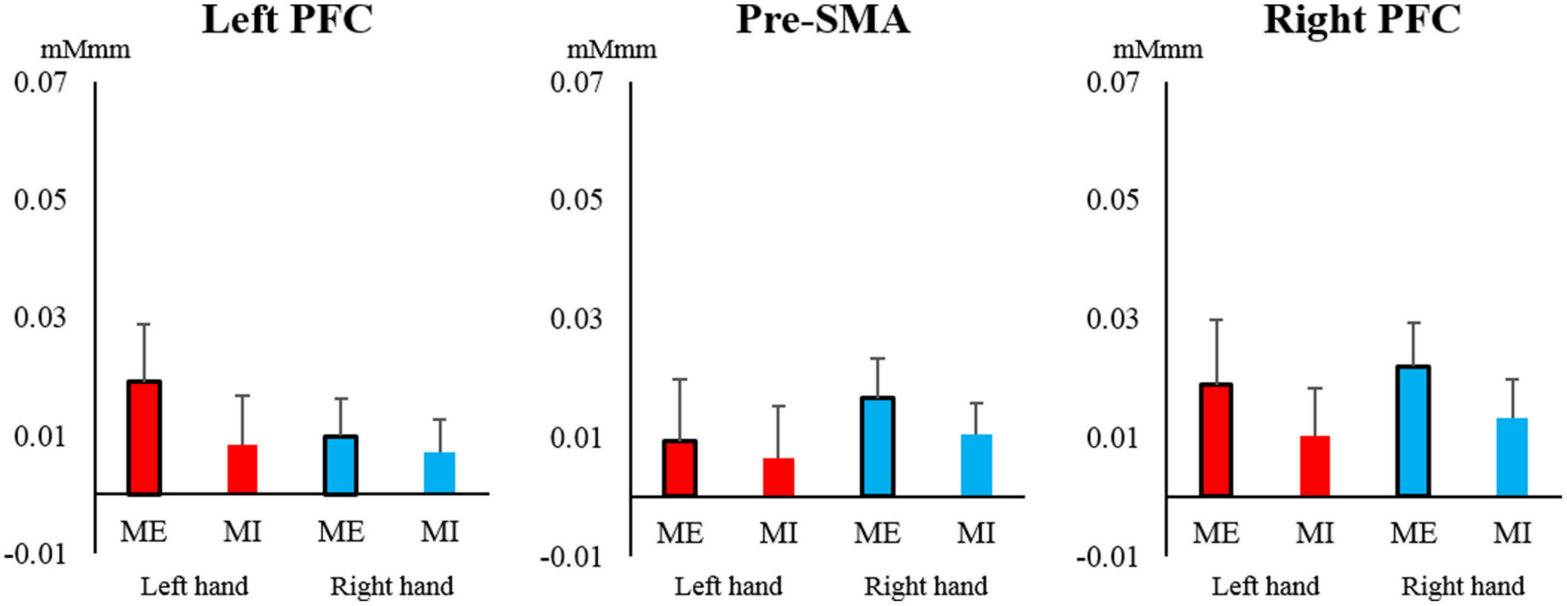
**Changes in oxy-Hb concentration during the task in the pre-supplementary motor area (pre-SMA) and prefrontal cortex (PFC)**. In the left and right PFC and pre-SMA, oxy-Hb showed relatively low total values and no significant main effects or interactions of task or hand (left hand, red; right hand, blue). Vertical bars represent the standard error.

In the PFC and pre-SMA, oxy-Hb showed a low total level, and no significant main effects or interactions were observed in the left PFC [task: *F*_(1, 19)_ = 0.030, *P* = 0.860, η^2^ = 0.000; hand: *F*_(1, 19)_ = 0.029, *P* = 0.866, η^2^ = 0.000; interaction: *F*_(1, 19)_ = 0.157, *P* = 0.696, η^2^ = 0.001], right PFC [task: *F*_(1, 19)_ = 1.166, *P* = 0.294, η^2^ = 0.013; hand: *F*_(1, 19)_ = 0.148, *P* = 0.705, η^2^ = 0.002; interaction: *F*_(1, 19)_ = 0.000, *P* = 1.000, η^2^ = 0.000], and pre-SMA [task: *F*_(1, 19)_ = 0.211, *P* = 0.651, η^2^ = 0.004; hand: *F*_(1, 19)_ = 0.537, *P* = 0.472, η^2^ = 0.006; interaction: *F*_(1, 19)_ = 0.047, *P* = 0.831, η^2^ = 0.001] (Figure 5). Most of the oxy-Hb concentration changes in the PFC and pre-SMA were only on the order of 0.01 mMmm, and the mean values were nearly identical for both tasks.

**Figure 6:**
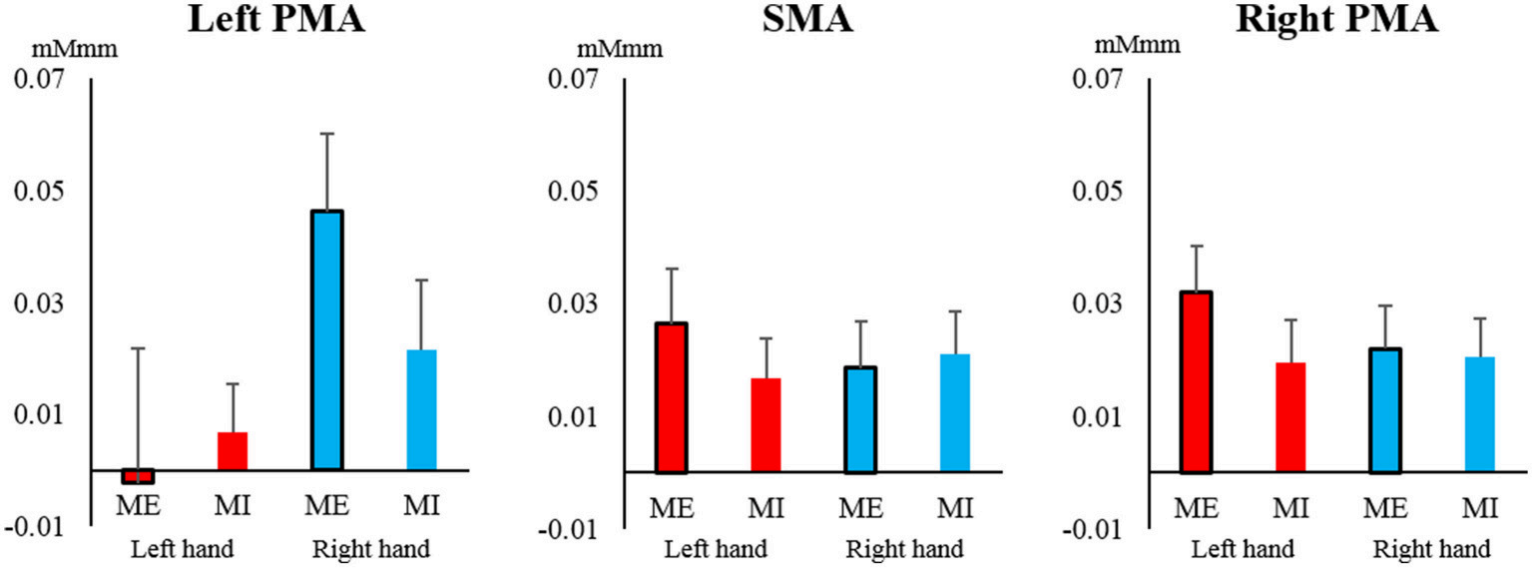
**Changes in oxy-Hb concentration during the task in the supplemental motor area (SMA) and pre-motor area (PMA)**. In the SMA and left and right PMA, the oxy-Hb level increased at a similar level, but there were no significant main effects or interactions of task or hand (left hand, red; right hand, blue). Vertical bars represent the standard error.

In contrast, in the SMA and PMA, the oxy-Hb concentration increased to a similar extent, and most of the observed changes were greater than 0.02 mMmm. However, no main effects or interactions were significant for the left PMA [task: *F*_(1, 19)_ = 0.260, *P* = 0.616, η^2^ = 0.003; hand: *F*_(1, 19)_ = 2.786, *P* = 0.111, η^2^ = 0.050; interaction: *F*_(1, 19)_ = 1.812, *P* = 0.194, η^2^ = 0.015], right PMA [task: *F*_(1, 19)_ = 1.145, *P* = 0.298, η^2^ = 0.011; hand: *F*_(1, 19)_ = 0.531, *P* = 0.475, η^2^ = 0.005; interaction: *F*_(1, 19)_ = 1.368, *P* = 0.257, η^2^ = 0.007], and SMA [task: *F*_(1, 19)_ = 0.165, *P* = 0.689, η^2^ = 0.002; hand: *F*_(1, 19)_ = 0.068, *P* = 0.798, η^2^ = 0.001; interaction: *F*_(1, 19)_ = 1.562, *P* = 0.227, η^2^ = 0.007; Figure 6].

**Figure 7:**
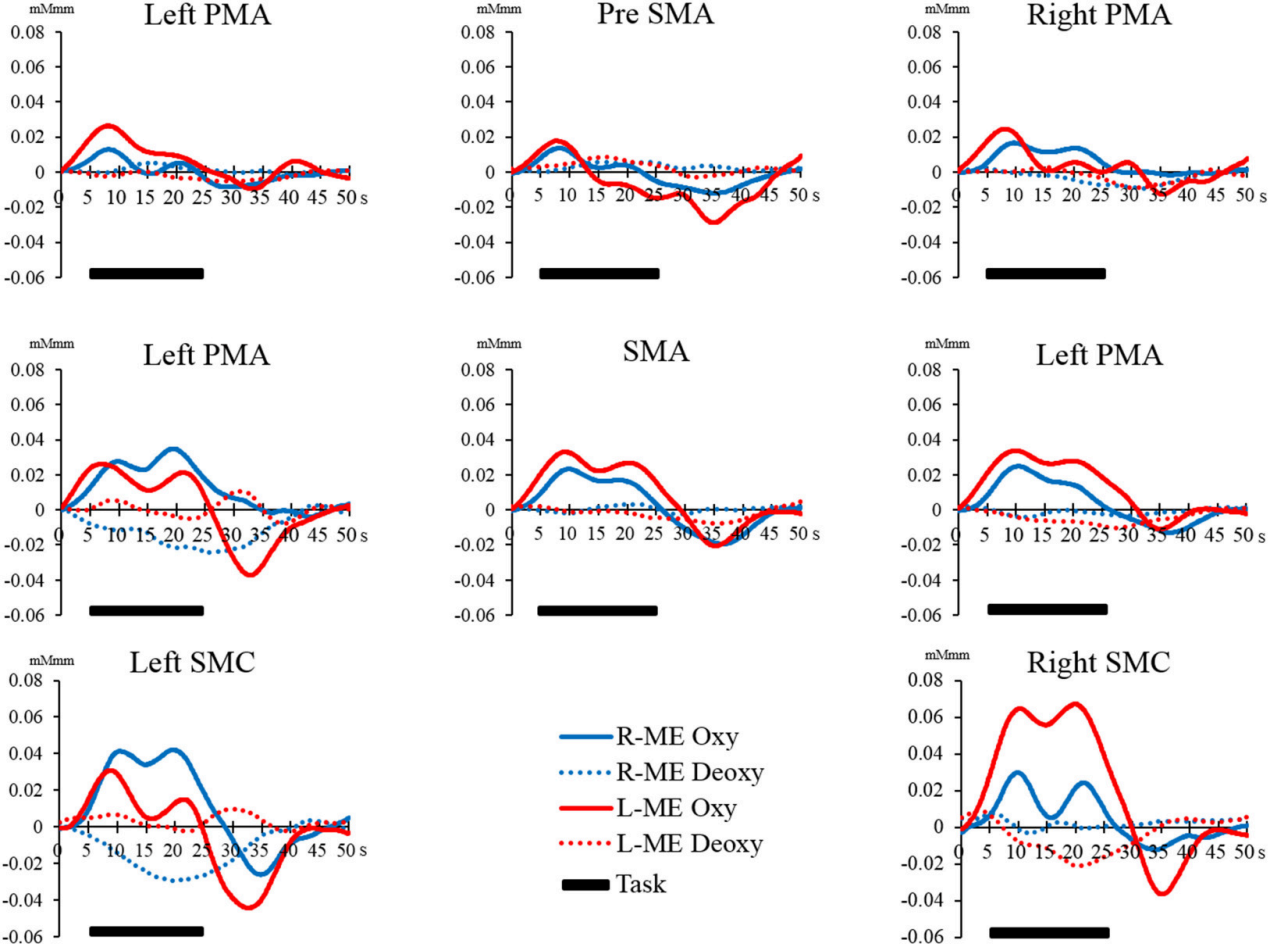
**Time course of changes in oxy-Hb and deoxy-Hb concentration during the ME task in each brain region**. The horizontal axis represents time course. The vertical axis represents changes in oxy-Hb and deoxy-Hb concentration. The solid line (left hand, red; right hand, blue) shows the oxy-Hb signal change. The dotted line shows the deoxy-Hb signal change. The horizontal bar represents the period during the task. The time course was averaged across all participants.

**Figure 8:**
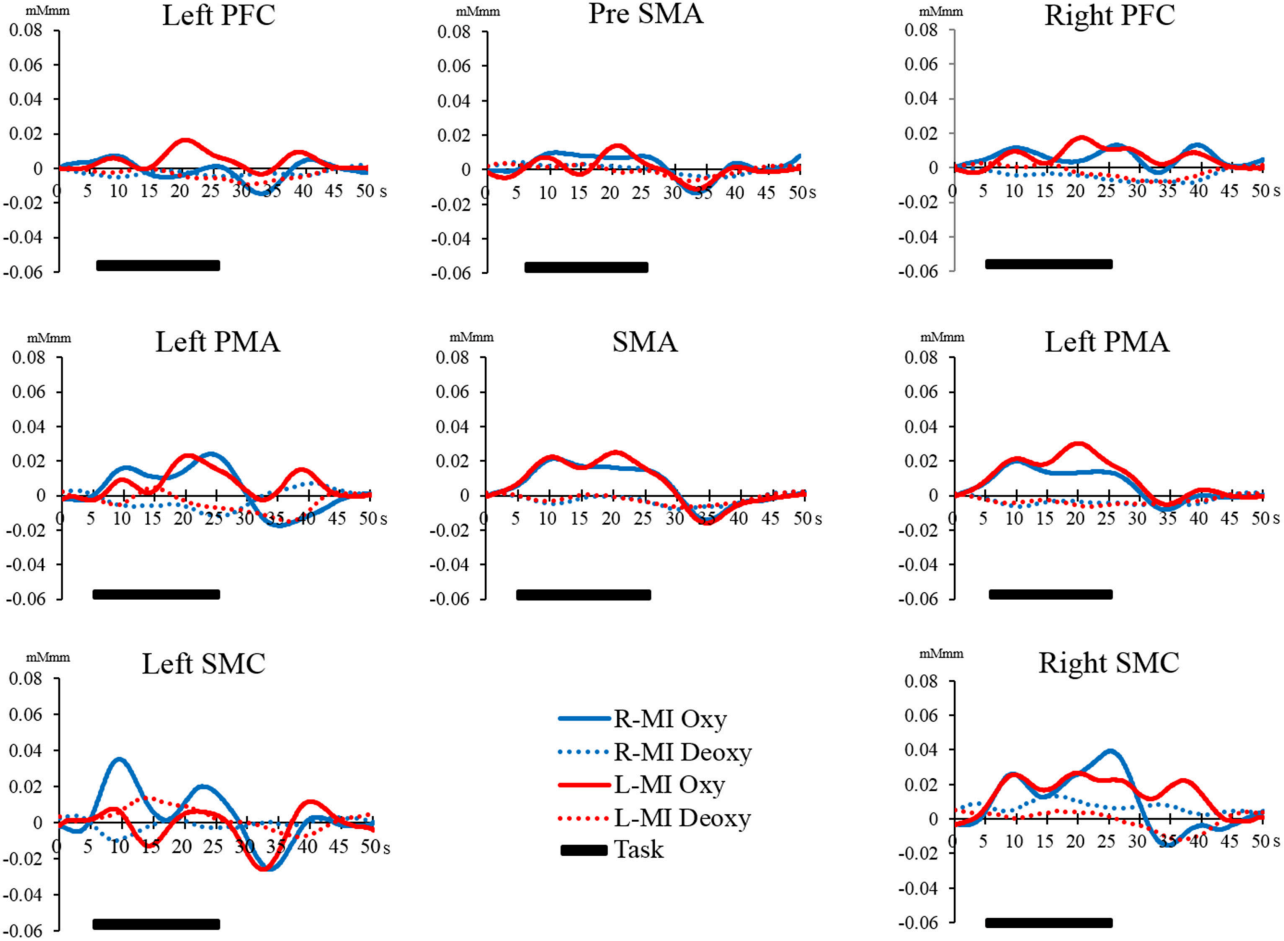
**Time course of changes in oxy-Hb and deoxy-Hb concentration during the MI task in each brain region**. The horizontal axis represents time course. The vertical axis represents changes in oxy-Hb and deoxy-Hb concentration. The solid line (left hand, red; right hand, blue) shows the oxy-Hb signal change. The dotted line shows the deoxy-Hb signal change. The horizontal bar represents the period during the task. The time course was averaged across all participants.

However, there were no significant differences in all regions of interest in the Bonferroni test. The interaction between the main effects and the effect sizes of the two factors of each brain region are presented in Table 1. The time courses of local regional hemodynamic changes of each task are shown in Figures 7, 8.

**Table 1:** **Statistic analysis of each the region of interest**.

**Region of interest**	**Hand**			**Task**			**Hand × Task**		
	***F*-value**	***P*-value**	**Effect size**	***F*-value**	***P*-value**	**Effect size**	***F*-value**	***P*-value**	**Effect size**
Left PFC	0.029	0.866	0.000	0.030	0.860	0.000	0.157	0.696	0.001
Right PFC	0.148	0.705	0.002	1.166	0.294	0.013	0.000	1.000	0.000
Pre SMA	0.537	0.472	0.006	0.211	0.651	0.004	0.047	0.831	0.001
Left PMA	2.786	0.111	0.050	0.260	0.616	0.003	1.812	0.194	0.015
Right PMA	0.531	0.475	0.005	1.145	0.298	0.011	1.368	0.257	0.007
SMA	0.068	0.798	0.001	0.165	0.689	0.002	1.562	0.227	0.007
Left SMC	5.590	0.029^*^	0.055	3.537	0.075	0.045	6.749	0.018^*^	0.058
Right SMC	3.658	0.071	0.036	3.795	0.066	0.030	5.146	0.035^*^	0.045

*Two-way ANOVA*,

* *p < 0.05*.

*Hand, Right hand × Left hand. Task, ME × MI*.

## Discussion

In this study, we clarified the hemodynamic signal changes in local regions by using NIRS during MI and ME of a tapping sequence task. In addition, we investigated the lateralization in brain activation between the right and left hand during ME and MI. To reliably distinguish MI from ME, we monitored muscle activation of the “tapping” hand during MI. Sufficient pre-experimental practice ensured that the participants performed the MI as vividly as possible, and NIRS measurements were started only when participants had practiced MI sufficiently.

### SMC activation

Oxy-Hb in our study showed significant interaction between task and hand in the SMC. This study showed particularly high activation of oxy- Hb in the ME of the contralateral hand. Previous imaging studies have shown that SMC activity directly relates to movement output (Obrig et al., 1996; Christensen et al., 2000) other studies showed increased activation of the primary motor cortex during MI with transcranial magnetic stimulation (TMS) (Lotze et al., 1999). Furthermore, some study using NIRS during MI have shown activation of the primary motor cortex.

However, some studies failed to demonstrate activation of the primary motor cortex (Hanakawa et al., 2003; Lotze et al., 2003; Cunnington et al., 2005). Wriessnegger et al. showed lower activation of primary motor areas during imagery than during execution in the study with NIRS. Our study, which used NIRS to measure hemodynamic signal changes in SMC, showed results similar to those of previous studies, confirming that we found the differences in the hemodynamic signal changes of the SMC measured using NIRS during MI and ME.

Further influence of handedness was found, in that, only the left SMC showed a main effect, in which the oxy-Hb level increased during ME of the right hand. This can be explained by the right-handedness of all participants. One study reported activation in the contralateral regions during ME and MI (Obrig et al., 1996), and another study showed that the SMC was activated more strongly during simple tasks (Lotze et al., 1999). Moreover, studies using TMS reported that activation of the primary motor cortex differed between the hemispheres, suggesting a functional asymmetry between the left and right hands (Yahagi and Kasai, 1999; Stinear et al., 2006).

The task we adopted in this study was a finger tapping sequence, which was quite intricate. Thus, the task was easier for the dominant hand than for the non-dominant hand, which may have caused the higher brain activation during ME and MI with the right hand.

### PFC and pre-SMA activation

Overall, changes in oxy-Hb concentration were low in the PFC and pre-SMA during both ME and MI. Some NIRS studies reported higher increase in oxy-Hb during cognitive tasks than during motor tasks (Toronov et al., 2001; Kakimoto et al., 2009), and some studies showed activation of the PFC even during MI (Ingvar and Philipson, 1977; Lacourse et al., 2005). MI is considered mental simulation with cognitive elements (Decety et al., 1988) that are expected to increase the activation of the PFC during MI.

However, the PFC did not show the expected activation during MI in this study. We suspect that the difficulty level of the task in this study might have been low, although it included intricate elements, which required the participants to practice the task in advance.

Similar to the PFC, it was previously reported that the pre-SMA is an important region for the early stages of motor learning (Nakamura et al., 1998), which is activated not only during ME but also during MI (Malouin et al., 2003). Thus, the reason why the pre-SMA was not activated during ME and MI in this study could be that the participants had sufficiently practiced the tasks in advance. In particular, pre-SMA activity reflects stages of motor learning (Decety et al., 1988). It has been reported that the pre-SMA is activated in the first stage of motor learning in every type of activity. When a person performs MI of an activity that has not been sufficiently learned, the vividness of the MI is low. The activation of the pre-SMA might be related to the vividness of MI, and this relationship should be further investigated using NIRS.

### SMA and PMA activation

In contrast to other regions, the SMA and PMA were activated to a similar extent during both tasks. These results were similar to those of previous studies that measured activation using PET or fMRI (Stephan et al., 1995; Hikosaka et al., 1996; Ruby and Decety, 2003; Solodkin et al., 2004).

It has been reported that the SMA is involved in movement planning and is activated not only during ME but also during the preparation and inhibition of movements (Tyszka et al., 1994; Kasess et al., 2008; Guillot et al., 2012). The SMA is also considered to be a major region that is activated during MI, and it has been shown to be closely related to ME and to become activated even during ME (Drenckhahn et al., 2015), which is different from the activation of the pre-SMA (Di Rienzo et al., 2014). As previously reported, in our study, SMA showed high activation during both ME and MI.

Some studies reported that the PMA was also activated during both tasks (Dechent et al., 2004), and that the effect of MI and subsequent PMA activation increased when participants were given feedback regarding the NIRS hemodynamic signal changes of the contralateral motor-related cortex, including the PMA (Mihara et al., 2012). Thus, the PMA has already been confirmed as an important domain for MI.

The finding that the SMA and PMA were similarly activated during both ME and MI is consistent with previous studies and suggests that the participants were able to vividly perform MI. Based on these findings, we infer that the hemodynamic signal changes in the SMA and PMA correspond to MI.

Therefore, based on the previous findings that SMA and PMA are responsible for MI, we believe that the vividness of MI might be visualized by measuring the activity of these regions. However, since we did not verify the vividness of MI directly in this study, further study will be needed to inspect the relationship between vividness of MI and SMA and PMA.

## Limitations

We adopted a tapping sequence task consisting of four conditions: ME and MI with the right and left hand. In this study, we observed hemodynamic signal changes during the tapping sequence task. However, as various types of motor imaging tasks were used in previous studies, the hemodynamic signal changes might be different depending on the task. Therefore, the same results cannot be guaranteed for other tasks. In addition, our study could not clarify the influence of the vividness of MI because our research directly investigated the vividness of MI.

Therefore, the aim of this study was to monitor hemodynamic signal changes of regions related to motor execution with NIRS during MI and ME by using a tapping sequence task, and to clarify topographical localization by motor-related regional hemodynamic signal changes during MI.

## Conclusions

Our study showed local hemodynamic signal changes of motor-related areas during MI, and, to our knowledge, this is the study localizing these changes during ME and MI by using NIRS. In particular, this study reports that SMA and PMA are similarly activated during both ME and MI.

It may be possible to evaluate the vividness of MI from the degree of activation of the SMA and PMA in future studies. However, further studies are needed to investigate the relationship between hemodynamic signal changes in these regions and subjective evaluation of MI vividness, which could reveal a potential approach for evaluating MI more objectively. We would additionally like to promote the development of effective mental practices that could contribute to the development of neuro-rehabilitation tools.

## Author contributions

Conceived and designed the experiments: NI, AS, TH. Performed the experiments: NI, TM, TH. Analyzed the date: NI, EK, YK, TT, TH. Wrote the paper: NI, TM, AS, FI, KT, TH.

### Conflict of interest statement

The authors declare that the research was conducted in the absence of any commercial or financial relationships that could be construed as a potential conflict of interest.

## References

[B1] Amemiya K. Ishizu T. Ayabe T. Kojima S. (2010). Effects of motor imagery on intermanual transfer: a near-infrared spectroscopy and behavioural study. Brain Res. 1343, 93–103. 10.1016/j.brainres.2010.04.048 20423702

[B2] Atsumori H. Kiguchi M. Obata A. Sato H. Katura T. Funane T.. (2009). Development of wearable optical topography system for mapping the prefrontal cortex activation. Rev. Sci. Instrum. 80, 043704. 10.1063/1.3115207 19405663

[B3] Christensen L. O. D. Johannsen P. Sinkjaer T. Petersen N. Pyndt H. S. Nielsen J. B. (2000). Cerebral activation during bicycle movements in man. Exp. Brain Res. 135, 66–72. 10.1007/s002210000493 11104128

[B4] Cooper R. J. Gagnon L. Goldenholz D. M. Boas D. A. Greve D. N. (2012). The utility of near-infrared spectroscopy in the regression of low-frequency physiological noise from functional magnetic resonance imaging data. Neuroimage 59, 3128–3138. 10.1016/j.neuroimage.2011.11.028 22119653 PMC3288700

[B5] Cope M. Delpy D. T. (1988). System for long-term measurement of cerebral blood and tissue oxygenation on newborn infants by near infra-red transillumination. Med. Biol. Eng. Comput. 26, 289–294. 10.1007/BF02447083 2855531

[B6] Cunnington R. Windischberger C. Moser E. (2005). Premovement activity of the pre-supplementary motor area and the readiness for action: studies of time-resolved event-related functional MRI. Hum. Mov. Sci. 24, 644–656. 10.1016/j.humov.2005.10.001 16337295

[B7] Decety J. Philippon B. Ingvar D. H. (1988). rCBF landscapes during motor performance and motor ideation of a graphic gesture. Eur. Arch. Psychiatry Neurol. Sci. 238, 33–38. 10.1007/BF00381078 3215218

[B8] Dechent P. Merboldt K.-D. Frahm J. (2004). Is the human primary motor cortex involved in motor imagery? Brain Res. Cogn. Brain Res. 19, 138–144. 10.1016/j.cogbrainres.2003.11.012 15019710

[B9] Di Rienzo F. Guillot A. Daligault S. Delpuech C. Rode G. Collet C. (2014). Motor inhibition during motor imagery: a MEG study with a quadriplegic patient. Neurocase 20, 524–539. 10.1080/13554794.2013.826685 23998364

[B10] Drenckhahn C. Koch S. P. Dümmler J. Kohl-Bareis M. Steinbrink J. Dreier J. P. (2015). A validation study of the use of near-infrared spectroscopy imaging in primary and secondary motor areas of the human brain. Epilepsy Behav. 49, 118–125. 10.1016/j.yebeh.2015.04.006 25976181

[B11] Fazli S. Mehnert J. Steinbrink J. Curio G. Villringer A. Müller K. R.. (2012). Enhanced performance by a hybrid NIRS-EEG brain computer interface. Neuroimage 59, 519–529. 10.1016/j.neuroimage.2011.07.084 21840399

[B12] Gregg M. Hall C. Butler A. (2010). The MIQ-RS: a suitable Option for examining movement imagery ability. Evid. Based Complement. Altern. Med. 7, 249–257. 10.1093/ecam/nem170 PMC2862926 18955294

[B13] Guillot A. Di Rienzo F. Macintyre T. Moran A. Collet C. (2012). Imagining is not doing but involves specific motor commands: a review of experimental data related to motor inhibition. Front. Hum. Neurosci. 6:247. 10.3389/fnhum.2012.00247 22973214 PMC3433680

[B14] Hanakawa T. Immisch I. Toma K. Dimyan M. A. Van Gelderen P. Hallett M. (2003). Functional properties of brain areas associated with motor execution and imagery. J. Neurophysiol. 89, 989–1002. 10.1152/jn.00132.2002 12574475

[B15] Hatakenaka M. Miyai I. Mihara M. Sakoda S. Kubota K. (2007). Frontal regions involved in learning of motor skill-A functional NIRS study. Neuroimage 34, 109–116. 10.1016/j.neuroimage.2006.08.014 17067821

[B16] Hikosaka O. Sakai K. Miyauchi S. Takino R. Sasaki Y. Pütz B. (1996). Activation of human presupplementary motor area in learning of sequential procedures: a functional MRI study. J. Neurophysiol. 76, 617–621. 8836248 10.1152/jn.1996.76.1.617

[B17] Hoshi Y. Kobayashi N. Tamura M. (2001). Interpretation of near-infrared spectroscopy signals: a study with a newly developed perfused rat brain model. J. Appl. Physiol. 90, 1657–1662. 11299252 10.1152/jappl.2001.90.5.1657

[B18] Ikeda K. Higashi T. Sugawara K. Tomori K. Kinoshita H. Kasai T. (2012). The effect of visual and auditory enhancements on excitability of the primary motor cortex during motor imagery: a pilot study. Int. J. Rehabil. Res. 35, 82–84. 10.1097/MRR.0b013e32834d2032 22002108

[B19] Ingvar D. H. Philipson L. (1977). Distribution of cerebral blood flow in the dominant hemisphere during motor ideation and motor performance. Ann. Neurol. 2, 230–237. 10.1002/ana.410020309 103483

[B20] Jeannerod M. (2001). Neural simulation of action: a unifying mechanism for motor cognition. Neuroimage 14, 103–109. 10.1006/nimg.2001.0832 11373140

[B21] Kakimoto Y. Nishimura Y. Hara N. Okada M. Tanii H. Okazaki Y. (2009). Intrasubject reproducibility of prefrontal cortex activities during a verbal fluency task over two repeated sessions using multi-channel near-infrared spectroscopy. Psychiatry Clin. Neurosci. 63, 491–499. 10.1111/j.1440-1819.2009.01988.x 19496994

[B22] Kasess C. H. Windischberger C. Cunnington R. Lanzenberger R. Pezawas L. Moser E. (2008). The suppressive influence of SMA on M1 in motor imagery revealed by fMRI and dynamic causal modeling. Neuroimage 40, 828–837. 10.1016/j.neuroimage.2007.11.040 18234512

[B23] Kimberley T. J. Khandekar G. Skraba L. L. Spencer J. A. Van Gorp E. A. Walker S. R. (2006). Neural substrates for motor imagery in severe hemiparesis. Neurorehabil. Neural Repair 20, 268–277. 10.1177/1545968306286958 16679504

[B24] Kober S. E. Wood G. (2014). Changes in hemodynamic signals accompanying motor imagery and motor execution of swallowing: a near-infrared spectroscopy study. Neuroimage 93, 1–10. 10.1016/j.neuroimage.2014.02.019 24576696

[B25] Kober S. E. Wood G. Kurzmann J. Friedrich E. V. C. Stangl M. Wippel T.. (2014). Near-infrared spectroscopy based neurofeedback training increases specific motor imagery related cortical activation compared to sham feedback. Biol. Psychol. 95, 21–30. 10.1016/j.biopsycho.2013.05.005 23714227

[B26] Koenraadt K. L. Roelofsen E. G. Duysens J. Keijsers N. L. (2014). Cortical control of normal gait and precision stepping: an fNIRS study. Neuroimage 85, 415–422. 10.1016/j.neuroimage.2013.04.070 23631980

[B27] Lacourse M. G. Orr E. L. R. Cramer S. C. Cohen M. J. (2005). Brain activation during execution and motor imagery of novel and skilled sequential hand movements. Neuroimage 27, 505–519. 10.1016/j.neuroimage.2005.04.025 16046149

[B28] Lareau E. Lesage F. Pouliot P. Nguyen D. Le Lan J. Sawan M. (2011). Multichannel wearable system dedicated for simultaneous electroencephalography/near-infrared spectroscopy real-time data acquisitions. J. Biomed. Opt. 16, 096014. 10.1117/1.3625575 21950928

[B29] Liu K. P. Chan C. C. Lee T. M. Hui-Chan C. W. (2004). Mental imagery for promoting relearning for people after stroke: a randomized controlled trial. Arch. Phys. Med. Rehabil. 85, 1403–1408. 10.1016/j.apmr.2003.12.035 15375808

[B30] Lotze M. Halsband U. (2006). Motor imagery. J. Physiol. 99, 386–395. 10.1016/j.jphysparis.2006.03.012 16716573

[B31] Lotze M. Montoya P. Erb M. Hülsmann E. Flor H. Klose U.. (1999). Activation of cortical and cerebellar motor areas during executed and imagined hand movements: an fMRI study. J. Cogn. Neurosci. 11, 491–501. 10.1162/089892999563553 10511638

[B32] Lotze M. Scheler G. Tan H.-R. M. Braun C. Birbaumer N. (2003). The musician's brain: functional imaging of amateurs and professionals during performance and imagery. Neuroimage 20, 1817–1829. 10.1016/j.neuroimage.2003.07.018 14642491

[B33] Malouin F. Richards C. L. Jackson P. L. Dumas F. Doyon J. (2003). Brain activations during motor imagery of locomotor-related tasks: a PET study. Hum. Brain Mapp. 19, 47–62. 10.1002/hbm.10103 12731103 PMC6872050

[B34] Malouin F. Richards L. C. Jackson L. P. Lafleur F. M. Doyon J. (2007). The Kinesthetic and Visual Imagery Questionnaire (KVIQ) for assessing motor imagery in persons with physical disabilities: a reliability and construct validity study. J. Neurol. Phys. Ther. 31, 20–29. 10.1097/01.NPT.0000260567.24122.64 17419886

[B35] Marumo K. Takizawa R. Kawakubo Y. Onitsuka T. Kasai K. (2009). Gender difference in right lateral prefrontal hemodynamic response while viewing fearful faces: a multi-channel near-infrared spectroscopy study. Neurosci. Res. 63, 89–94. 10.1016/j.neures.2008.10.012 19056435

[B36] Mehagnoul-Schipper D. J. van der Kallen B. F. W. Colier W. N. J. M. van der Sluijs M. C. van Erning L. J. T. O. Thijssen H. O. M.. (2002). Simultaneous measurements of cerebral oxygenation changes during brain activation by near-infrared spectroscopy and functional magnetic resonance imaging in healthy young and elderly subjects. Hum. Brain Mapp. 16, 14–23. 10.1002/hbm.10026 11870923 PMC6871837

[B37] Mihara M. Miyai I. Hattori N. Hatakenaka M. Yagura H. Kawano T.. (2012). Neurofeedback using real-time near-infrared spectroscopy enhances motor imagery related cortical activation. PLoS ONE 7:e32234. 10.1371/journal.pone.0032234 22396753 PMC3292558

[B38] Miyai I. Yagura H. Hatakenaka M. Oda I. Konishi I. Kubota K. (2003). Longitudinal optical imaging study for locomotor recovery after stroke. Stroke 34, 2866–2870. 10.1161/01.STR.0000100166.81077.8A 14615624

[B39] Mulder T. Zijlstra S. Zijlstra W. Hochstenbach J. (2004). The role of motor imagery in learning a totally novel movement. Exp. Brain Res. 154, 211–217. 10.1007/s00221-003-1647-6 14508635

[B40] Nakamura K. Sakai K. Hikosaka O. (1998). Neuronal activity in medial frontal cortex during learning of sequential procedures. J. Neurophysiol. 80, 2671–2687. 9819272 10.1152/jn.1998.80.5.2671

[B41] Obrig H. Hirth C. Junge-Hülsing J. G. Döge C. Wolf T. Dirnagl U.. (1996). Cerebral oxygenation changes in response to motor stimulation. J. Appl. Physiol. 81, 1174–1183. 8889751 10.1152/jappl.1996.81.3.1174

[B42] Obrig H. Villringer A. (2003). Beyond the visible—imaging the human brain with light. J. Cereb. Blood Flow Metab. 23, 1–18. 10.1097/01.WCB.0000043472.45775.29 12500086

[B43] Ohno K. Higashi T. Sugawara K. Ogahara K. Funase K. Kasai T. (2011). Excitability changes in the human primary motor cortex during observation with motor imagery of chopstick use. J. Phys. Ther. Sci. 23, 703–706. 10.1589/jpts.23.703

[B44] Okamoto M. Dan H. Sakamoto K. Takeo K. Shimizu K. Kohno S.. (2004). Three-dimensional probabilistic anatomical cranio-cerebral correlation via the international 10–20 system oriented for transcranial functional brain mapping. Neuroimage 21, 99–111. 10.1016/j.neuroimage.2003.08.026 14741647

[B45] Oldfield R. C. (1971). The assessment and analysis of handedness: the Edinburgh inventory. Neuropsychologia 9, 97–113. 10.1016/0028-3932(71)90067-4 5146491

[B46] Page S. J. Levine P. Sisto S. Johnston M. V. (2001). A randomized efficacy and feasibility study of imagery in acute stroke. Clin. Rehabil. 15, 233–240. 10.1191/026921501672063235 11386392

[B47] Page S. J. Murray C. Hermann V. Levine P. (2012). Retention of motor changes in chronic stroke survivors who were administered mental practice. Arch. Phys. Med. Rehabil. 92, 1741–1745. 10.1016/j.apmr.2011.06.009 21880300 PMC3257856

[B48] Pelgrims B. Michaux N. Olivier E. Andres M. (2011). Contribution of the primary motor cortex to motor imagery: a subthreshold TMS study. Hum. Brain Mapp. 32, 1471–1482. 10.1002/hbm.21121 21077146 PMC6870368

[B49] Piper S. K. Krueger A. Koch S. P. Mehnert J. Habermehl C. Steinbrink J.. (2014). A wearable multi-channel fNIRS system for brain imaging in freely moving subjects. Neuroimage 85, 64–71. 10.1016/j.neuroimage.2013.06.062 23810973 PMC3859838

[B50] Pu S. Nakagome K. Yamada T. Yokoyama K. Matsumura H. Mitani H.. (2012). The relationship between the prefrontal activation during a verbal fluency task and stress-coping style in major depressive disorder: a near-infrared spectroscopy study. J. Psychiatr. Res. 46, 1427–1434. 10.1016/j.jpsychires.2012.08.001 22935269

[B51] Riccio I. Iolascon G. Barillari M. R. Gimigliano R. Gimigliano F. (2010). Mental practice is effective in upper limb recovery after stroke: a randomized single-blind cross-over study. Eur. J. Phys. Rehabil. Med. 46, 19–25. 20332722

[B52] Roland P. E. Larsen B. Lassen N. A. Skinhøj E. (1980). Supplementary motor area and other cortical areas in organization of voluntary movements in man. J. Neurophysiol. 43, 118–136. 7351547 10.1152/jn.1980.43.1.118

[B53] Ruby P. Decety J. (2003). What you believe versus what you think they believe: a neuroimaging study of conceptual perspective-taking. Eur. J. Neurosci. 17, 2475–2480. 10.1046/j.1460-9568.2003.02673.x 12814380

[B54] Sagari A. Iso N. Moriuchi T. Ogahara K. Kitajima E. Tanaka K.. (2015). Changes in cerebral hemodynamics during complex motor learning by character entry into touch-screen terminals. PLoS ONE 10:e0140552. 10.1371/journal.pone.0140552 26485534 PMC4618511

[B55] Schuster C. Hilfiker R. Amft O. Scheidhauer A. Andrews B. Butler J.. (2011). Best practice for motor imagery: a systematic literature review on motor imagery training elements in five different disciplines. BMC Med. 9:75. 10.1186/1741-7015-9-75 21682867 PMC3141540

[B56] Sharma N. Pomeroy V. M. Baron J. C. (2006). Motor imagery: a backdoor to the motor system after stroke? Stroke 37, 1941–1952. 10.1161/01.STR.0000226902.43357.fc 16741183

[B57] Solodkin A. Hlustik P. Chen E. E. Small S. L. (2004). Fine modulation in network activation during motor execution and motor imagery. Cereb. Cortex 14, 1246–1255. 10.1093/cercor/bhh086 15166100

[B58] Steinkellner O. Gruber C. Wabnitz H. Jelzow A. Steinbrink J. Fiebach J. B.. (2010). Optical bedside monitoring of cerebral perfusion: technological and methodological advances applied in a study on acute ischemic stroke. J. Biomed. Opt. 15, 061708. 10.1117/1.3505009 21198156

[B59] Stephan K. Fink G. Passingham R. Silbersweig D. Ceballosbaumann A. Frith C.. (1995). Functional-anatomy of the mental representation of upper extremity movements in healthy-subjects. J. Neurophysiol. 73, 373–386. 7714579 10.1152/jn.1995.73.1.373

[B60] Stinear C. M. Byblow W. D. (2003). Motor imagery of phasic thumb abduction temporally and spatially modulates corticospinal excitability. Clin. Neurophysiol. 114, 909–914. 10.1016/S1388-2457(02)00373-5 12738438

[B61] Stinear C. M. Fleming M. K. Byblow W. D. (2006). Lateralization of unimanual and bimanual motor imagery. Brain Res. 1095, 139–147. 10.1016/j.brainres.2006.04.008 16713588

[B62] Tobias J. D. (2006). Cerebral oxygenation monitoring: near-infrared spectroscopy. Expert Rev. Med. Devices 3, 235–243. 10.1586/17434440.3.2.235 16515389

[B63] Toronov V. Webb A. Choi J. H. Wolf M. Michalos A. Gratton E.. (2001). Investigation of human brain hemodynamics by simultaneous near-infrared spectroscopy and functional magnetic resonance imaging. Medical Phys. 28, 521–527. 10.1118/1.1354627 11339749

[B64] Tyszka J. M. Grafton S. T. Chew W. Woods R. P. Colletti P. M. (1994). Parceling of mesial frontal motor areas during ideation and movement using functional magnetic resonance imaging at 1.5 tesla. Ann. Neurol. 35, 746–749. 10.1002/ana.410350617 8210233

[B65] Wriessnegger S. C. Kurzmann J. Neuper C. (2008). Spatio-temporal differences in brain oxygenation between movement execution and imagery: a multichannel near-infrared spectroscopy study. Int. J. Psychophysiol. 67, 54–63. 10.1016/j.ijpsycho.2007.10.004 18006099

[B66] Yahagi S. Kasai T. (1999). Motor evoked potentials induced by motor imagery reveal a functional asymmetry of cortical motor control in left- and right- handed human subjects. Neurosci. Lett. 276, 185–188. 10.1016/S0304-3940(99)00823-X 10612636

